# Self-Efficacy to Regulate Eating Behaviors Scale for Children: A Validation Study

**DOI:** 10.3390/ijerph20042807

**Published:** 2023-02-04

**Authors:** Cátia Silva, Beatriz Pereira, Gabriela Figueiredo, Pedro Rosário, José Carlos Núñez, Paula Magalhães

**Affiliations:** 1 Department of Applied Psychology, University of Minho, 4710-052 Braga, Portugal; 2 Department of Psychology, University of Oviedo, 33003 Oviedo, Spain

**Keywords:** self-efficacy, self-regulation, eating behavior, food temptations, negative emotions, children, validation

## Abstract

Self-efficacy has a strong influence on children’s eating behavior. Feeling capable of regulating one’s eating behavior is especially relevant in situations of activation while facing temptations or experiencing negative emotions. Despite the relevance, there is no validated measure to assess children’s self-efficacy to regulate eating behaviors in these domains. The present study examines the psychometric properties of the Self-Efficacy to Regulate Eating Behaviors Scale for Children based on a sample of 724 elementary school children in Portugal. The sample was split randomly into two groups, and a principal component analysis with Group 1 and a confirmatory factor analysis with Group 2 were carried out. The scale comprises two distinct but related factors—self-efficacy to regulate eating behaviors in activation and temptation situations and self-efficacy to regulate eating behaviors in negative emotional situations. Moreover, self-efficacy to regulate eating behaviors was positively and statistically related to self-regulation processes toward healthy eating, declarative knowledge about healthy eating, and attitudes and perceptions toward healthy eating. The present study provides preliminary evidence that the Self-Efficacy to Regulate Eating Behaviors Scale for Children is valid and reliable for evaluating children’s self-efficacy in regulating their eating behaviors.

## 1. Introduction

Healthy eating behavior is considered a public health priority for preventing chronic diseases (e.g., obesity, diabetes, cancer) across all ages [1]. Following a healthy and balanced diet during childhood has been identified as a primary factor for good health across the entire life span [1]. Thus, promoting and improving healthy eating in children may be a particularly effective approach to attaining healthy lives and diminishing chronic diseases in future generations.

In recent years, the literature has highlighted the relevance of motivational-related factors in children’s adoption and maintenance of healthy eating behaviors [2,3]. Among several motivational-related factors, self-efficacy is one of the leading contributors to children’s healthy eating [4,5]. Self-efficacy can be understood as the individual’s belief in their ability to perform a particular behavior or reach a specific goal successfully, including adopting healthy eating [6,7]. 

Considering the importance of self-efficacy for children adopting and maintaining healthy eating, there is a lack of validated and children-focused measures of self-efficacy for healthy eating behaviors. Self-efficacy measures in the eating behavior domain for the general population are scarce and target mainly disordered eating and obesity [8,9]. In particular, these scales focused on individuals’ self-efficacy for eating draw on a restrictive vision of the phenomenon, i.e., control eating behavior by canceling an individual’s immediate response to food [10,11]. To the best of the authors’ knowledge, only the scale developed by Lasseter and colleagues [12]: The Healthy Eating and Physical Activity Self-Efficacy Questionnaire (HEPASEQ-C) follows a promotional approach. Overall, the results showed that this tool has acceptable validity and internal consistency and is appropriate for upper elementary school children and health promotion settings [12]. However, this scale focuses on self-efficacy regarding generic daily eating behaviors (e.g., I will eat at least four servings of vegetables every day) rather than on the belief in one’s ability to proactively self-regulate eating behavior during challenging situations (e.g., when facing temptations and negative emotions [13,14]). Research has pointed out that high self-efficacy beliefs aid children to regulate and engage in healthy eating when facing temptations or experiencing negative affect [13,14,15,16]. Self-efficacy impacts eating behavior indirectly through self-regulation strategies [4,6]; therefore, developing an eating self-efficacy scale that considers children’s ability to regulate eating behaviors is needed. 

As previously mentioned, currently, there is a lack of validated scales assessing children’s self-efficacy to regulate eating behaviors, explicitly targeting the contextual factors related to these behaviors [6]. A study developing and evaluating a scale focused on self-efficacy for regulating eating behaviors would be valuable to help understand the differential influence of specific situations in which children usually struggle to stick to healthy eating. Following this need, the Social Cognitive Theory [17] set the ground for the current work. According to this theoretical framework, self-efficacy measures must target a specific domain, its related factors, and a particular population. To better assess this construct, Bandura [6] published guidelines that should be considered when constructing such scales. Overall, these guidelines state that scales should include items reflecting several situations in which individuals may find it challenging to perform a specific behavior (in our case, healthy eating behaviors, e.g., watching TV or going to the supermarket) and several items per behavior/domain to cover distinct aspects of the domain [6].

The present study aims to evaluate the psychometric properties of the Self-Efficacy to Regulate Eating Behaviors scale for Children (SEREB-C). This scale focused on two dimensions likely to challenge children’s healthy food choices: activation and temptation situations and negative emotional situations [13,14]. Current purposes are: (a) to examine the SEREB-C’s factor structure by conducting a principal component analysis (PCA) with one half of the sample and confirmatory factor analysis (CFA) with the second half of the sample; (b) to assess its reliability (i.e., Cronbach’s alpha and omega coefficients); and (c) to assess its validity evidence regarding the relationship with three external measures—self-regulation processes towards healthy eating, declarative knowledge about healthy eating, and attitudes and perceptions towards healthy eating. Prior research shows that self-regulated behavior is predicted by high self-efficacy beliefs [4]; therefore, we hypothesized that the SEREB-C is positively associated with self-regulation processes toward healthy eating. Furthermore, as knowledge and attitudes are likely to influence eating behavior positively [4,18], we hypothesized that the SEREB-C is positively associated with declarative knowledge of, and attitudes and perceptions towards, healthy eating. 

## 2. Materials and Methods

### 2.1. Participants

Children were recruited from 6 Portuguese public schools from different environments (i.e., rural and urban). A total of 827 elementary school children (*n* = 61 classes) from the fifth and sixth grades were invited to participate in the current investigation. From this pool of participants, 103 parents/legal guardians and children did not consent or assent to participate. Thus, the final sample was composed of 724 children (49% girls) aged between 8 and 13 years (M = 10.58; SD = 0.71).

### 2.2. Procedure

The present study was part of a broader investigation approved by the University of Minho Ethics Committee for Research in Social and Human Sciences (CEICSH) (CEICSH 032/2019). Elementary school children and their parents or legal guardians were informed about the study’s aims and assured of the data’s confidentiality. Afterward, parents or legal guardians of the participants provided written informed consent and children provided assent to participate. Before data collection, researchers participated in a training session hosted by a senior researcher to set the protocol for data collection. Researchers administered the instruments (discussed below) during regular classes as follows: children were invited by the researcher to fill in the instruments and were asked to complete the task by themselves; each child fulfilled the instruments at their own pace, and was supported by the researcher in items found to be unclear. When in doubt about an item, that sentence was explained to the whole class similarly. The children took approximately 30 min to complete the instruments in Qualtrics XM survey platform^®^ version 2020 [19]. 

### 2.3. Instruments

#### 2.3.1. Self-Efficacy to Regulate Eating Behaviors Scale for Children (SEREB-C)

Bandura [6] developed a guide for constructing self-efficacy scales, providing examples of sample scales on distinct spheres of self-efficacy (e.g., eating habits, exercise, pain management). Based on a sample scale provided in this guide, we developed the SEREB-C, presented in the Portuguese language. The present scale comprises 14 items assessing children’s perceived capability to regulate the choice of healthy food in challenging daily situations. While developing this scale, we queried several self-efficacy and healthy eating promotion experts to check whether the daily situations presented in the items were appropriate for children. Based on their inputs, we removed the items related to scenarios that were only adequate for adults (e.g., preparing meals for others) or specific to restrictive diets (e.g., parties where much appetizing high-fat food is served). Additionally, we added a new item related to a scenario often reported as a child’s barrier to healthy eating (i.e., when I see unhealthy but appealing food at the school cafeteria or vending machines) [14,20]. As displayed in Table 1, SEREB-C assessed self-efficacy in scenarios related to (a) activation and temptation situations, such as having much unhealthy food at home (8 items), and (b) negative emotional situations, such as when children are feeling upset or worried with school issues (6 items). 

SEREB-C was introduced with the following indication: “A number of situations are described below that can make it hard to stick to a healthy diet. For each sentence, please select the answer that best represents how certain you are that you can stick to a healthy diet on a regular basis” (see Appendix A, Table A1). Each item was scored on a 5-point Likert scale, where 1 stands for “I am sure that I am not capable of choosing a healthy option,” 2 for “I think I may not be capable of choosing a healthy option,” 3 for “I think sometimes I am capable, other times I am not capable of choosing a healthy option,” 4 for “I think I may be capable of choosing a healthy option,” and 5 for “I am sure that I am capable of choosing a healthy option.” Items were formatted positively to reduce the likelihood of response bias [21]. The points of the individual items were summed to create a composite score ranging from 8 to 40 for the activation and temptation situations subscale and from 6 to 30 for the negative emotional situations subscale. Higher scores implied greater self-efficacy in regulating eating behaviors in the corresponding situations. For the present investigation, the correlations among the 8 items from the activation and temptation situations varied from 0.42 to 0.61, and the reliability of this subscale was good (α = 0.82; ω = 0.82; EVA = 0.37; RC = 0.82). The correlations among the 6 items from the negative emotional situations varied from 0.57 to 0.72, and the reliability of this subscale was good (α = 0.86; ω = 0.86; EVA = 0.51; RC = 0.86).

#### 2.3.2. Self-Regulation Processes toward Healthy Eating

Self-regulation was assessed using the Self-Regulation Processes towards Healthy Eating Questionnaire [22]. The scale comprised 9 items regarding the participant’s use of self-regulation strategies toward healthy eating (e.g., “I plan my meals. I think about what I am going to eat and what it takes to prepare my meal—for example, after waking up, I think about what I will eat for breakfast and what I need to prepare it”; “I try to apply in my daily life the information on healthy eating that I receive at home, at school, at the health center or elsewhere.”). Participants’ responses were scored on a Likert-like scale ranging from 1 (never) to 5 (always) and summed to create a composite score ranging from 9 to 45, with higher scores implying higher use of self-regulation strategies. The reliability of the scale is good (α = 0.83; ω = 0.82; AVE = 0.40; CR = 0.85).

#### 2.3.3. Declarative Knowledge about Healthy Eating

Declarative knowledge about healthy eating was assessed using an adapted version of the Knowledge of Healthy Eating Questionnaire [23]. The scale consisted of 10 statements, and children rated their agreement regarding each (e.g., “our meal should contain varied and colorful foods”). In the original instrument, items were scored from 1 (totally disagree) to 5 (totally agree) in a Likert-like format [23]. In the present study, responses were scored as true or false. The correct answers were summed to create a composite score ranging from 0 to 10, with higher scores implying more declarative knowledge about healthy eating. The reliability of this scale is moderate (KR-20 = 0.66).

#### 2.3.4. Attitudes and Perceptions towards Healthy Eating

Attitudes and perceptions towards healthy eating were assessed using an adapted version of the Students’ Attitudes and Perceptions on Healthy Eating Questionnaire [4]. During data collection, children stated that an item from the original scale (i.e., drinking water is not very important for my health; [4]) was not clear (due to the negative wording). Thus, after discussion, the research team decided to withdraw this item from the questionnaire. Thus, the current scale comprised 16 statements about children’s attitudes and perceptions regarding the importance of healthy eating (e.g., eating fruit and vegetables will help me to grow up). In the present study, responses were scored on a Likert-like scale ranging from 1 (totally disagree) to 5 (totally agree). Responses were summed to create a composite score ranging from 16 to 80, with higher scores implying more positive attitudes and perceptions toward healthy eating. The reliability of this scale is very good (α = 0.83; ω = 0.82; AVE = 0.36; CR = 0.89).

### 2.4. Data Analysis

The data were analyzed in several phases, following the purposes of the present study. First, missing values for the 14 items of SEREB-C ranged from 0.1% to 1.0% (M = 0.55%) and were imputed using regression imputation. Second, to examine the SEREB-C’s factor structure, participants were randomly split into two groups (i.e., Group 1, *n* = 357; Group 2, *n* = 367).

With Group 1, we conducted a principal component analysis (PCA) using SPSS, version 28.0, with direct oblimin rotation (delta = 0). The appropriate number of factors for retention was determined by several criteria: the scree plots, eigenvalue > 1.0, and conceptual meaningfulness of items on each factor.

With Group 2, we conducted a confirmatory factor analysis (CFA) using AMOS, version 28.0. Aiming to examine whether self-efficacy to regulate eating behaviors in activation and temptation situations and self-efficacy to regulate eating behaviors in negative emotional situations were empirically distinguishable, we compared the difference in goodness-of-fit between (a) a one-factor model (i.e., factorially indistinct) and (b) a two-factor model (i.e., factorially distinct). Moreover, the models were evaluated through multiple goodness-of-fit indicators, including CFI ≥ 0.95 [24]; TLI ≥ 0.95 [24]; RMSEA ≤ 0.05 indicating good fit and RMSEA ≤.08 indicating reasonable fit [25,26]; and SRMR < 0.08 [24]. Additionally, we used the Akaike information criterion (AIC) to compare alternative models as it considers both the goodness-of-fit and the number of parameters [27]; typically, smaller values indicate a better fit [24].

The SEREB-C’s reliability (i.e., convergent validity) was assessed using average variance extracted, composite reliability, and alpha and omega coefficients. According to Hair et al. [28], average variance extracted values equal to or greater than 0.50 and lower than composite reliability indicates adequate convergent validity. However, when the average variance extracted is lower than 0.5, but composite reliability is higher than 0.6, the convergent validity of the construct can also be considered adequate [29]. The criterion of α ≥ 0.70 was used to determine the adequacy of the alpha coefficient for research purposes [30]. Additionally, we followed the recommendation that the adequacy of the omega coefficient needs to meet the same criterion as the alpha coefficient [31]. Finally, Pearson correlations between the SEREB-C and 3 external measures were examined to analyze concurrent and predictive validity. In particular, the associations between the SEREB-C and self-regulation processes toward healthy eating, declarative knowledge about healthy eating, and attitudes and perceptions toward healthy eating were assessed.

## 3. Results

### 3.1. Principal Component Analysis (PCA)

For Group 1 (*n* = 357), the Kaiser–Meyer–Olkin measure of sampling adequacy indicated that this group was appropriate for PCA (KMO = 0.915). PCA results revealed a two-factor solution accounting for 52.23% of the total variance. All items loaded rather acceptably (>0.50) on the two factors, which can be appropriately referred to as (1) activation and temptation situations, and (2) negative emotional situations. Table 1 presents the factor pattern and structure coefficients. 

### 3.2. Confirmatory Factor Analysis (CFA)

For Group 2 (*n* = 367), CFA findings showed that, compared with the one-factor solution (MLRχ2 = 350.038; df = 77; CFI = 0.863; TLI = 0.838; RMSEA = 0.098; 90% CI [0.088–0.109]; SRMR = 0.062), the two-factor solution yielded a much better fit to the data (MLRχ2 = 259.125; df = 76; CFI = 0.908; TLI = 0.890; RMSEA = 0.081; 90% CI [0.070–0.092]; SRMR = 0.0536) (see Table 2). Therefore, self-efficacy to regulate eating behaviors in activation and temptation situations and self-efficacy to regulate eating behaviors in negative emotional situations were empirically distinguishable for Group 2. 

Each of the 14 items was specified to load on only one factor in the two-factor solution (i.e., either activation and temptation situations or negative emotional situations); therefore, the structure coefficients estimated indicator–construct correlations [32]. As displayed in Table 3, the standardized estimates for each of the 14 indicators are acceptable (ranging from 0.484 to 0.799), providing additional empirical support for convergent validity [33]. Moreover, the average variance extracted and composite reliability values provided empirical support for convergent validity. Finally, the estimated correlation between activation and temptation situations and negative emotional situations was 0.617, *p* < 0.001. 

### 3.3. Reliability

The means of the scale for the two groups combined (*n* = 724) were 3.61 (SD = 0.83) for activation and temptation situations and 3.28 (SD = 1.02) for negative emotional situations. The alpha coefficient for activation and temptation situations was 0.82, the corresponding omega coefficient was 0.82, the alpha coefficient for negative emotional situations was 0.86, and the corresponding omega coefficient was 0.86. These reliability coefficients were considered good in measurement practice [30,31]. Item–total correlations for SEREB-C varied from 0.418 to 0.689, indicating good homogeneity.

### 3.4. Concurrent and Predictive Validity

Table 4 shows the Pearson correlation coefficients of the relationships between the two factors of SEREB-C and three relevant external measures (i.e., self-regulation processes towards healthy eating, declarative knowledge about healthy eating, and attitudes and perceptions towards healthy eating). Results confirmed the hypothesis that both factors were positively and statistically related to self-regulation processes toward healthy eating, declarative knowledge about healthy eating, and attitudes and perceptions toward healthy eating.

## 4. Discussion

The present study aimed to validate the SEREB-C. Our preliminary results indicated that SEREB-C has good psychometric quality regarding reliability (i.e., exhibits good Cronbach’s alpha and omega coefficients) and validity evidence (e.g., positive relationship with external relevant measures). Moreover, the two-factor model was a better fit than the one-factor model. In the current study, items focused on activation and temptation situations were saturated in one factor, and items focused on negative emotional situations were saturated in the other. The activation and temptation situations factor describes triggers that make children struggle to cope with unhealthy foods and to make healthy choices (e.g., school breaks and having much unhealthy food at home) [14]. In contrast, the negative emotional situations factor describes circumstances that can make children use food as a comfort, e.g., to regulate their emotions when feeling bored or worried [34]. For example, many children consider homework boring and pointless, which may create a feeling of tension [35,36,37].

Regarding concurrent and predictive validity, our preliminary results confirmed the hypothesis that the SEREB-C is positively related to self-regulation processes towards healthy eating, declarative knowledge about healthy eating, and attitudes and perceptions toward healthy eating [4,18,22,38]. Pearson correlation coefficients were higher for the activation and temptation situations factor than for the negative emotional situations factor. Moreover, Pearson correlations were low for declarative knowledge about healthy eating and moderate for self-regulation processes and attitudes and perceptions toward healthy eating. These findings are consistent with recent research showing a positive relationship between self-efficacy for, self-regulation towards, knowledge about, and attitudes towards healthy eating, with knowledge being the less contributing factor for eating behaviors [4,39]. Thus, these findings stressed that through coping with situations of activation, temptation, and negative emotions, self-efficacy to regulate eating behaviors might play an essential role in children adopting healthy eating behaviors. 

Health professionals, teachers, and counselors could use SEREB-C to evaluate children’s self-efficacy to regulate their healthy eating behaviors and design tailored interventions to support the development of positive self-efficacy beliefs to regulate eating behaviors accordingly. Research has been suggesting that the design of interventions should not only transmit knowledge about healthy eating but combine it with training on self-regulation strategies related to healthy eating behaviors [22,40]. In fact, interventions that promote self-regulation in healthy eating are among the most effective and are likely to show long-lasting results [40]. These interventions could comprise, for example, activities promoting goal-setting, self-monitoring, and evaluation of self-consequences [40,41]. Finally, SEREB-C could also be used to assess the impact of these interventions on the healthy eating domain. Regarding future directions, researchers may also use SEREB-C to extend the study of the relationships between children’s self-efficacy to regulate eating behaviors and other variables likely to influence their food consumption (e.g., perceived social support [42]). Despite the promising contributions of the current study, we would like to stress some limitations, such as the sample being composed only of children from the fifth and sixth grades. Including a representative sample of elementary school children from the first to the sixth grade would be valuable. Moreover, further research is needed to validate the SEREB-C in other cultures, as self-efficacy to regulate eating behaviors could be culturally sensitive [43] and may impact children’s eating behaviors following cultural settings (e.g., prohibition of vending machines at school or school-based initiatives to promote fruit and vegetable consumption) [43,44].

## 5. Conclusions

In the present study, the factor structure of the SEREB-C was examined using exploratory and confirmatory factor analysis. Our preliminary results showed that SEREB-C comprises two factors: activation and temptation situations and negative emotional situations. The two-factor model showed a good fit for Portuguese elementary school children. The scale also had good reliability coefficients. Thus, SEREB-C can be used by practitioners and researchers to assess children’s self-efficacy to regulate their eating behaviors. Moreover, SEREB-C values can provide valuable information to design tailored interventions. Finally, the psychometric properties of SEREB-C should be further explored with samples of different cultures.

## Figures and Tables

**Table 1:** Rotated Factor Pattern (Structure) Matrix for the SEREB-C *.

		Factor	
Item		1	2
1	While I am watching TV	0.761 (0.723)	
2	During school breaks	0.590 (0.682)	
3	When I am very hungry	0.454 (0.504)	
4	When I have much unhealthy food at home	0.577 (0.668)	
5	When I am happy	0.783 (0.762)	
6	When I am physically active and feel energized	0.903 (0.789)	
7	When I see unhealthy but appealing food in the supermarket	0.553 (0.649)	
8	When I see unhealthy but appealing food at the school bar or vending machines	0.462 (0.576)	
9	When I feel restless or upset		−0.601 (0.684)
10	When I feel upset or worried about school stuff		−0.756 (0.781)
11	When I am angry		−0.909 (0.841)
12	When I am sad		−0.836 (0.817)
13	While doing homework		−0.471 (0.667)
14	When I get bored with family and friends		−0.677 (0.752)

Note: Group 1 (*n* = 357). * SEREB-C = Self-Efficacy to Regulate Eating Behaviors for Children.

**Table 2:** Model Comparison: Summary of Goodness-of-Fit Indices.

Models	MLRχ^2^	Df	CFI	TLI	RMSEA	RMSEA 90% CI	SRMR	AIC
One-factor model	350.038	77	0.863	0.838	0.098	0.088–0.109	0.0614	406.038
Two-factor model	259.125	76	0.908	0.890	0.081	0.070–0.092	0.0536	317.125

Note: Group 2 (*n* = 367); CFI = Comparative Fit Index; TLI = Tucker–Lewis Index; RMSEA = Root Mean Square Error of Approximation; CI = Confidence Interval; SRMR = Standard Root Mean Squared Residual; AIC = Akaike Information Criterion.

**Table 3:** Standardized Coefficients for the Two-Factor CFA Model.

Latent Construct	Item	β
Activation and temptation situations	1	0.613
	2	0.643
	3	0.598
	4	0.574
	5	0.670
	6	0.608
	7	0.651
	8	0.484
Negative emotional situations	9	0.799
	10	0.726
	11	0.734
	12	0.736
	13	0.654
	14	0.630

Note: Group 2 (*n* = 367); Composite reliability (CR) for activation and temptation situations = 0.82; Average variance extracted (AVE) for activation and temptation situations = 0.37; Composite reliability (CR) for negative emotional situations = 0.86; Average variance extracted (AVE) for negative emotional situations = 0.51.

**Table 4:** Pearson correlations between SEREB-C and the 3 external measures and mean, and standard deviation of the external measures.

	SEREB-C			
External Measures	Activation and Temptation Situations	Negative Emotional Situations	M	SD
Self-regulation processes toward healthy eating	0.546 ***	0.461 ***	33.093	6.252
Declarative knowledge about healthy eating	0.126 ***	0.119 ***	8.905	1.506
Attitudes and perceptions towards healthy eating	0.465 ***	0.408 ***	62.863	8.865

Note: SEREB-C = Self-Efficacy to Regulate Eating Behaviors for Children; *n* = 724; *** *p* < 0.001; Self-regulation processes toward healthy eating: min. = 9, max. = 45; Declarative knowledge about healthy eating: min. = 0, max. = 10; Attitudes and perceptions towards healthy eating: min. = 16, max. = 80.

## Data Availability

Data are available from the corresponding author upon reasonable request.

## Appendix A

**Table A1:** A number of situations are described below that can make it hard to stick to healthy eating. Please, for each sentence, select the answer that best represents how certain you are that you can stick to a healthy eating diet on a regular basis.

	1. I Am Sure That I Am not Capable of Choosing a Healthy Option	2. I Think I May Not be Capable of Choosing a Healthy Option	3. I Think Sometimes I Am Capable, Other Times I Am not Capable of Choosing a Healthy Option	4. I Think I May be Capable of Choosing a Healthy Option	5. I Am Sure That I Am Capable of Choosing a Healthy Option
1. While I am watching TV					
2. During school breaks					
3. When I am very hungry					
4. When I have much unhealthy food at home					
5. When I am happy					
6. When I am physically active and feel energized					
7. When I see unhealthy but appealing food in the supermarket					
8. When I see unhealthy but appealing food at the school bar or vending machines					
9. When I feel restless or upset					
10. When I feel upset or worried about school stuff					
11. When I am angry					
12. When I am sad					
13. While doing homework					
14. When I get bored with family and friends					
